# Minutes of the Proceedings of the National Medical Convention

**Published:** 1846-09

**Authors:** 


*Minutes of the Proceedings of the National Medical Convention,* held in the city of New York, in May last.

Pursuant to a call of the New York State Medical Society, a Convention composed of delegates from medical colleges, and societies of many of the states of the union, was held in the Hall of the Medical Department of the University of the city of New York, on the 5th and 6th of May, 1846. Thirty-three colleges and medical societies were represented on the occasion, as appears from the published minutes of the proceedings, and there were one hundred and nineteen delegates in attendance.

Dr. J. Knight, of New Haven, was elected President of the Convention, and Drs. John Bell, of Philadelphia, and Edward Delafield, of New York city, Vice-Presidents; Drs. Richard D. Arnold, of Savannah, and Alfred Stille, of Philadelphia, were appointed Secretaries.

The convention, after a session of two days, during which time various resolutions were passed, and a number of committees appointed, adjourned to meet in Philadelphia, on the first Wednesday in May, 1847.—*Balt. Ed.*

